# Rietveld Structure Refinement of Cu-Trien Exchanged Nontronites

**DOI:** 10.3389/fchem.2018.00558

**Published:** 2018-11-20

**Authors:** Xiaoli Wang, Libing Liao

**Affiliations:** ^1^Institute of Mineralogy, TU Bergakademie Freiberg, Freiberg, Germany; ^2^Key Laboratory of Materials Utilization of Nonmetallic Minerals and Solid Wastes, National Laboratory of Mineral Materials, School of Materials Science and Technology, China University of Geosciences, Beijing, China

**Keywords:** nontronites, Rietveld refinement, Cu-triethylenetetramine, crystal structure, X-ray powder diffraction

## Abstract

The Rietveld analysis of X-ray powder diffraction patterns is used widely for obtaining the structural information of clay minerals. However, the complex hydration behavior and the variability of interlayer contents are often considered difficult to be described correctly by a simple structure model. In the present work, the use of Cu-triethylenetetramine (Cu-trien)-exchanged nontronites has been proposed to simplify the interlayer structure. This method provides a potential to obtain the structural information of nontronites, for example, the layer charge density, occupancies of *cis*-octahedral sites, and the iron content by the Rietveld analysis from the X-ray powder diffraction patterns. The approach was demonstrated on three Cu-trien-exchanged nontronite samples. The Rietveld refinements were carried out first on the purified samples and the results showed a good peak fitting between measured and calculated patterns. The refined iron content and the occupancies of *cis*-octahedral sites are in general agreement with the reference data, which have been obtained from chemical and thermal analyses. The refinement of layer charge density showed lower values compared with the reference. It may be due to the assumption of temperature factor of Cu-trien in the interlayer. A raw sample with natural impurities was chosen to test the applicability of this method. The refinement pattern of the raw sample led to good agreement with the observed data. The results of the iron content and the occupancies of *cis*-octahedral sites showed the same tendency as purified samples. This study showed that this approach allows for obtaining some structural details of nontronites directly from X-ray powder diffraction patterns of Cu-trien-exchanged samples.

## Introduction

Nontronite is the iron-rich dioctahedral smectite. The dominant cation in the tetrahedral sheet is Si^4+^, which can be substituted by Al^3+^ commonly (Manceau et al., 2000a). The octahedral sheet contains predominantly Fe^3+^, partially Al^3+^, and a minor amount of Mg^2+^. Only two-thirds of the octahedral positions are occupied by cations in dioctahedral smectites. In general, octahedral sheet shows two different configurations, that is, *cis*- and *trans*-octahedron that relate to the disposition of hydroxyl groups. In the *cis*-octahedron, the OH groups are on the same side, whereas in the *trans*-octahedron, the OH groups are on the opposite side. Tsipursky and Drits (1984) found that natural dioctahedral smectites may cover a wide range of *cis*-vacant (*cv*) and *trans*-vacant (*tv*) proportions. Based on the oblique-texture electron diffraction and X-ray diffraction analyses, Besson et al. (1983) and Tsipursky and Drits (1984) demonstrated that for Fe-rich dioctahedral smectites, *cis*-octahedral positions were occupied and *trans*-octahedral sites were vacant. In general, nontronites show turbostratic stacking disorder (Biscoe and Warren, 1942) due to the weak bonds between the 2:1 layers (Moore and Reynolds, 1997). This kind of disorder can be described when the layers rotate or translate randomly to each other along the c^*^ direction (Moore and Reynolds, 1997). This kind of structural defect leads to non-Bragg diffraction effects and restricts the applicability of the conventional Rietveld method (Bish, 1993) to smectite. Several attempts of the Rietveld refinement have been done on turbostratically disordered structure (Taylor and Matulis, 1994; Viani et al., 2002; Gournis et al., 2008). However, these authors assumed more or less the ordered structure models, but not a real turbostratically disordered structure. The BGMN software can describe turbostratic disorder features of the diffraction patterns successfully by using the structure model containing a single-layer approach (Ufer et al., 2004). This method allowed an acceptable quantification of the smectite content in bentonites (Ufer et al., 2008). Later, it was applied for the Rietveld refinement of illite-smectite mixed-layer minerals (Ufer et al., 2012). However, this approach cannot handle the complex hydration behavior in the interlayer (Sato et al., 1992, 1996; Ferrage et al., 2005b).

Sposito et al. (1999) concluded the hydration shells of the cations like Na^+^ and K^+^ and the results indicated a tendency of inhomogeneous distribution in the interlayer of montmorillonite. Ferrage et al. (2005a,b) showed the hydration behavior of the smectites and the configuration of the interlayer to be complex and varying. It seems that the correct description of the interlayer configuration is another difficulty for the Rietveld analysis of nontronites, except for the turbostratically disordered structure. Therefore, a defined and stable occupancy of the interlayer space, which is independent of humidity, may provide a potential for the modeling of such modified interlayer structures. In general, the intercalation of ethylene glycol (EG) in smectites is used for the characterization of smectites and vermiculites (MacEwan and Wilson, 1984). However, EG is not sufficiently stable for long-time measurements. The Cu-triethylenetetramine (Cu-trien) is a kind of stable complex and used routinely in the determination of the cation exchange capacity (CEC) (Meier and Kahr, 1999). The high selectivity of the index cation [Cu(trien)]^2+^makes the exchange with the interlayer content fast and complete (Bergaya et al., 2006). Kaufhold et al. (2011) investigated the swelling capacity of Cu-trien-exchanged smectites and concluded that the Cu-trien-exchanged smectites showed constant *d*_001_ spacing and without significant water uptake. It may offer a chance to obtain information on the layer charge density by refining the occupancy of the Cu-trien complex in the interlayer spacing.

The current work applied X-ray diffraction analysis to the Cu-trien-exchanged nontronites by using the Rietveld method. The main objectives of this study are given as follows: (I) to investigate if some structural details of nontronites such as the layer charge density, iron content, and the occupancies of the *trans-* and *cis-* octahedral sites can be obtained directly from the X-ray diffraction patterns of purified samples and (II) to test the applicability of this method to raw nontronite samples with natural impurities.

## Materials and methods

### Sample preparation

**Figure 1:**
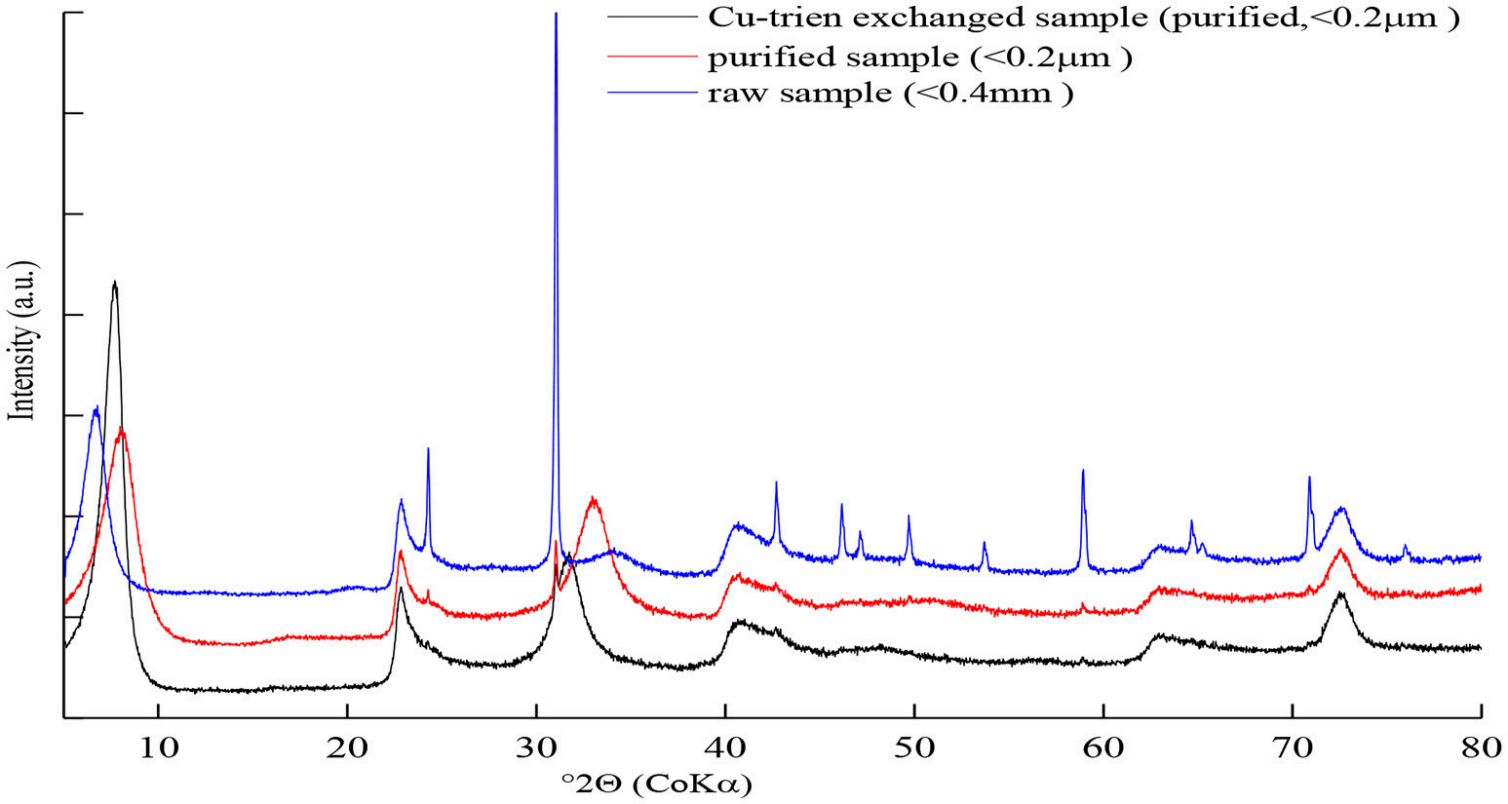
The XRD patterns of the sample NWa with different cation exchange states.

Two of the studied samples are from Uley Mine, South Australia (NAu-1 and NAu-2, Source Clays Repository) (Keeling et al., 2000). The other one is Washington nontronite (NWa). Keeling et al. (2000) found that both NAu-1 and NAu-2 showed high purity. This was also proved by primary X-ray diffraction analysis of samples. Thus, no further chemical treatment was performed for samples, NAu-1 and NAu-2, to remove impurities like carbonite, iron oxides, or organic matter. In contrast, sample NWa contained amounts of quartz and trace amounts of goethite (Figure 1 and Table 5). Due to the large size, quartz can be easily removed from samples by the particle size separation process. The common method for the dissolution of iron oxides is described by Mehra and Jackson (1960). However, Manceau et al. (2000a,b) found that more than 99% octahedral Fe^3+^ was reduced to Fe^2+^ after the removal of iron oxides by using this method. Also, the layer charge may also change during the reduction processes (Carrado et al., 2006). Therefore, this procedure was not applied on this sample to avoid the destruction of the structure. To get the enrichment of nontronites, the particle-size separation of the < 0.2 μm fraction from sodium-saturated samples was necessary. The excess salts were removed by dialysis and the < 0.2 μm fraction was obtained by centrifugation. Then, the purified samples (< 0.2 μm) were exchanged with Cu-trien complex: 0.8 g purified samples (< 0.2 μm) were suspended in 100 ml 0.1 M Cu-trien solution. After shaking for 24 h, the suspension was centrifuged and washed with deionized water. Then, 100 ml of fresh Cu-trien solution was added into the centrifuge tube and shaken for 3 h again to ensure the exchange reaction was completed. After washing, the dispersion was dialyzed to remove excess salts and then dried at room temperature.

The chemical composition of Cu-trien-exchanged nontronite samples (Table 1) was calculated from the analysis of XRF (Table 2) using the method described by Köster (1977) (Table 1). The XRF measurement was performed on a MagiX PRO XRF-spectrometer (stimulation power: 3.2 KW) in the Institute of Geosciences, the Johannes Gutenberg University, Mainz. Sample NAu-2 showed higher iron content and lower aluminum content compared with sample NAu-1 (Table 1). The result is in good agreement with the study of Keeling et al. (2000). The other sample, NWa, showed significantly lower iron content and higher aluminum than NAu-1 and NAu-2 (Table 1). The layer charge density was determined by the alkyl ammonium method (AAM) (Lagaly and Weiss, 1976; Olis et al., 1990; Lagaly, 1991, 1994). The occupancies of *cis*- and *trans*-octahedral sites were calculated by fitting the derivative thermogravimetry (dTG) curves, which provided the information of the dehydroxylation temperature (Drits et al., 1995, 1998). The results showed that almost all *cis*-octahedral sites were occupied (Table 1) and the octahedral sheets were dominated by *trans*-vacant structure. The occupancy of iron cations in the octahedral sites was obtained by dividing the octahedral iron content by the octahedral occupancy (Table 1). The structural information obtained from the chemical and thermal analyses was used as independent references to evaluate the Rietveld refinements.

**Table 1:** Structural formulae and properties of nontronite samples (< 0.2 μm fraction).

**Samples**	**Chemical composition /FU**	**Layer charge density [eq/FU]**	**Iron content per cation position**	**Occupancy of *cis*-octahedral sites**
Nau-1	${\text{Cu}}_{\text{0.18}}^{2+}\left({\text{Al}}_{0.21}{\text{Fe}}_{1.83}^{3+}\right)\left[\left({\text{Si}}_{3.51}{\text{Al}}_{0.49}\right){\text{O}}_{10}{\left(\text{OH}\right)}_{2}\right]$	0.37^*^	0.897	1
Nau-2	${\text{Cu}}_{0.12}^{2+}\left({\text{Al}}_{0.04}{\text{Fe}}_{1.94}^{3+}\right)\left[\left({\text{Si}}_{3.81}{\text{Al}}_{0.19}\right){\text{O}}_{10}{\left(\text{OH}\right)}_{2}\right]$	0.25^**^	0.980	1
NWa	${\text{Cu}}_{0.17}^{2+}\left({\text{Al}}_{0.43}{\text{Fe}}_{1.46}^{3+}{\text{Mg}}_{0.21}\right)\left[\left({\text{Si}}_{3.58}{\text{Al}}_{0.42}\right){\text{O}}_{10}{\left(\text{OH}\right)}_{2}\right]$	0.34^*^	0.695	0.955

* *Layer charge density according to Lagaly (1994)*.

** *Mean layer charge density according to Olis et al. (1990)*.

**Table 2:** XRF analysis of the Cu-trien-exchanged nontronites (< 0.2 μm).

**Oxides/%**	**SiO_2_**	**Al_2_O_3_**	**Fe_2_O_3_(t)**	**MnO**	**MgO**	**CaO**	**Na_2_O**	**TiO_2_**	**P_2_O_5_**	**CuO**	**LOI**
NAu1	52.04	8.78	36.08	0.01	0.00	0.03	0.00	0.03	0.00	4.56	17.46
NAu2	56.44	2.91	38.14	0.00	0.01	0.05	0.00	0.05	0.00	3.58	15.86
NWa	56.33	10.46	27.86	0.02	2.00	0.03	0.00	0.72	0.03	3.60	19.4

### X-ray powder diffraction analysis

The X-ray powder diffraction patterns of nontronites were collected by using a URD 6 (Seifert, CoKα radiation) diffractometer with a secondary beam graphite monochromator, a 0.2-mm detector slit, and an automatic divergence slit (15-mm irradiated length). The measurements were performed from 5° to 80° 2θ, with a step width of 0.03° 2θ and 3–5 s/step. The patterns were analyzed by the Analyze RayfleX v.2.352 software. The dried samples (< 0.2 μm) were ground in an agate mortar by hand and filled into the sample holder by using a side-loading technique. To reduce the influence of the preferred orientation on X-ray powder diffraction patterns, a specific method for sample preparation was applied. The powders passed through a sieve with 100-μm mesh to destroy the aggregates, which had formed during the previous drying process and formed a rough surface.

### Rietveld refinements

The program BGMN (Bergmann et al., 1998) was applied for the Rietveld refinement. The single-layer approach (Ufer et al., 2004) was used to describe the turbostratically disordered structure of nontronites. A standard cell was elongated 10 times in the stacking direction and filled only by one single 2:1 layer (Ufer et al., 2004). The atomic coordinates of the 2:1 layers of nontronites were taken from the Manceau et al. (2000a) study and recalculated for an orthogonal unit cell (Table 3). The positions of the atoms were kept constant during the refinement. The start value for the lattice constant *b* was set at 0.906 nm and refined with the limits ranging from 0.90 to 0.93 nm, and the lattice constant *a* was connected to *b* setting as $a\;=b\;/\;\sqrt{3}$ due to the assumption of the hexagonal layer symmetry (Manceau et al., 2000a). The start value of lattice constant *c* of Cu-trien-exchanged nontronites was set at 1.31 nm and refined with the limitation between 1.29 and 1.36 nm (Kaufhold et al., 2011). The atomic structure of the Cu-trien complex was derived from the Keramidas and Rentzeperis (1992) study. The Cu-trien molecule was placed in the middle of the interlayer and rotated parallel to the TOT layer. During the refinement, the atomic positions were kept fixed. The lattice constants were set as *a* = 0.7362 nm, *b* = 1.4708 nm, and *c* = 1.5551 nm (Keramidas and Rentzeperis, 1992). According to the study of Szczerba and Ufer (2018), the temperature factor of Cu-trien cation in the interlayer was set at 0.3 nm^2^, which should be significantly higher than the atoms in other positions.

**Table 3:** Atomic coordinates of the orthogonalized unit cell for nontronite recalculated from Manceau et al. (2000a).

	**x**	**y**	**z**
T1	0.63	0.329	0.78
T2	0	0.329	0.22
M1	0.316	0.333	0.5
M2	0.316	0	0.5
O1	0.63	0.688	0.613
O2	0.64	0.5	0.822
O3	0.375	0.736	0.845
O4	0.003	0.313	0.387
O5	0.97	0.5	0.179
O6	0.26	0.74	0.156
O7	0.683	0	0.611
O8	0.944	0	0.389
Na1	0.646	0	0.892
Na2	0	0	0.108
T1	0.63	0.671	0.78
T2	0	0.671	0.22
M1	0.316	0.667	0.5
O1	0.63	0.312	0.613
O3	0.375	0.264	0.845
O4	0.003	0.687	0.387
O6	0.26	0.26	0.156
T1	0.13	0.829	0.78
T2	0.5	0.829	0.22
M1	0.816	0.833	0.5
M2	0.816	0.5	0.5
O1	0.13	0.188	0.613
O2	0.14	0	0.822
O3	0.875	0.236	0.845
O4	0.503	0.813	0.387
O5	0.47	0	0.179
O6	0.76	0.24	0.156
O7	0.183	0.5	0.611
O8	0.444	0.5	0.389
Na1	0.146	0.5	0.892
Na2	0.5	0.5	0.108
T1	0.13	0.171	0.78
T2	0.5	0.171	0.22
M1	0.816	0.167	0.5
O1	0.13	0.812	0.613
O3	0.875	0.764	0.845
O4	0.503	0.187	0.387
O6	0.76	0.76	0.156

*Space group = C1/m1, a = 0.523 nm; b = 0.906 nm; c = 0.967 nm*.

*“M” indicates the cations Al^3+^, Mg^2+^, and Fe^3+^ in the octahedral sites*.

*“T” indicates the cations Si^4+^ and Al^3+^ in the tetrahedral sites*.

The occupancy of the Cu-trien complex in the interlayer P (Cu_trien_) and the iron occupation in the octahedral position P (Fe) were refined. The layer charge density was presented by the parameter P (Cu_trien_). Despite the nontronites being known to prefer the *trans*-vacant octahedral configuration, both *cis*- and *trans*-vacant sites are considered. The *cis*-site occupation P (*cis*-sites) was tried to be refined for checking this precondition. The scaling factor and the peak-broadening parameters were refined. The correction function for the preferred orientation was introduced and refined during the refinement. The zero-point shift correction and the sample displacement error were refined as nonstructural parameters.

## Results and discussion

### Rietveld refinement on purified Cu-trien-exchanged samples

The X-ray diffraction patterns of sample NWa with different treatments are shown in Figure 1. The patterns of the raw and purified Na-saturated sample displayed asymmetric and broad 001 reflection (Figure 1), which indicated complex hydration states and inhomogeneous layer charge distribution in the interlayer (Ferrage et al., 2005b, 2007). Thus, such a peak profile is difficult to be simulated by a simple structure model. After exchanging the interlayer contents with the Cu-trien, the intensity of the 001 reflection was enhanced and well-defined (Figure 1). This method makes the description of the interlayer structure much easier and provides a probability to obtain the layer charge density by refining the occupancy of the Cu-trien complex in the interlayer.

**Figure 2:**
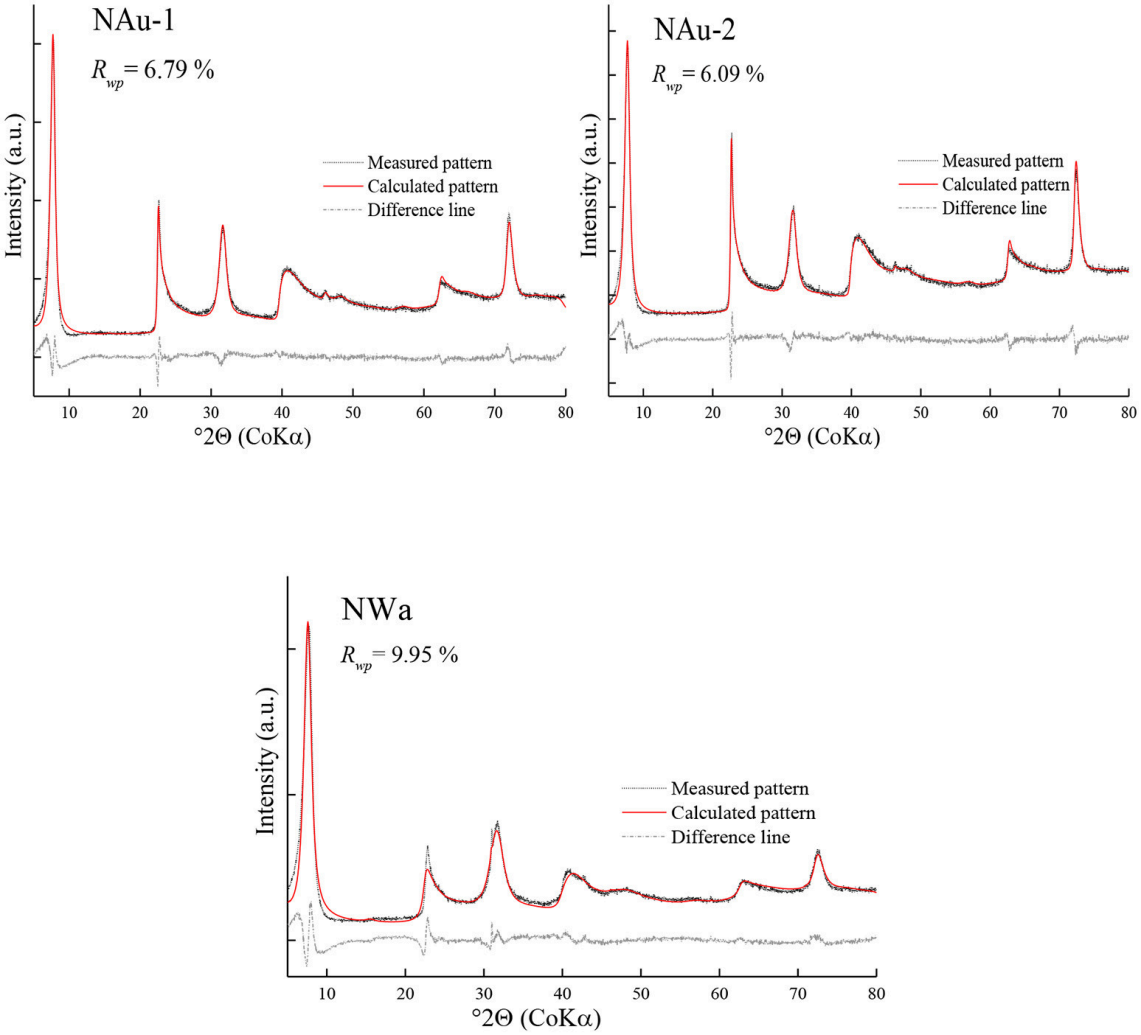
Rietveld refinements of purified nontronite samples.

The refinement of the sample NAu-1 showed a good agreement between the measured and calculated patterns and gave reasonable results for the iron content and the *cis-*octahedral sites occupancies (Table 4). The value of *R*_*wp*_ was 6.79% (Figure 2). The refined value of P (Fe) = 0.86(8) agreed with the chemical data within the estimated confidence interval (Table 4). The *cis*-octahedral site occupancy P (*cis*-sites) = 0.97(2) was close to the expected value of 1. On the contrary, the occupancy of Cu-trien in the interlayer P (Cu_trien_) = 0.312(5) was underestimated compared with the value 0.37 obtained by the AAM method (Table 4).

**Table 4:** The refinement results of the purified nontronite samples (< 0.2 μm).

			**Nau-1**		**Nau-2**		**Nwa**	
	**Start value**	**Refinement limits**	**Reference values**	**Refinement result *R_*wp*_* = 6.79% *R_*exp*_* = 2.63%**	**Reference values**	**Refinement result *R_*wp*_* = 6.09% *R_*exp*_* = 2.63%**	**Reference values**	**Refinement result *R_*wp*_* = 9.95% *R_*exp*_* = 2.68%**
**Contents/wt.%**								
Nontronite/[3σ]				100		100		99.4(2)
Quartz/[3σ]								0.1(2)
Goethite/[3σ]								0.5(6)
**Lattice parameter**								
*b*(nm)/[3σ]	0.906	0.90-0.93		0.914(1)		0.9101(2)		0.9072(2)
**Structure parameters**								
P(Fe)/[3σ]	0.4	0-1	0.897	0.86(8)	0.980	0.91(7)	0.695	0.65(9)
P(*cis*-sites)/[3σ]	0.5	0-1	1	0.97(2)	1	1	0.995	1
Layer charge density P(Cu_trien_)/[3σ]	0.2	0-0.4	0.37	0.312(5)	0.25	0.238(4)	0.34	0.310(6)
Preferred orientation/(correction factor in < 00*l*>, deviation from random orientation)	0			1.37(2)		1.23(2)		1.81(3)

*Reference values: P (Fe) from chemical analysis, P(cis-sites) from thermal analysis, P(Cu-trien) from layer charge density determined by the AAM method*.

The refinement of NAu-2 resulted in satisfactory results. The *R*_*wp*_ value was 6.09%. The iron content P (Fe) = 0.91(7) was near the value 0.980 as calculated from the structural formula. The refined values of P (Cu_trien_) = 0.238(4) and P (*cis*-sites) = 1 were in good agreement with the reference data (Table 4).

The refinement of the sample NWa got the *R*_*wp*_ value of 9.95% (Figure 2). Mismatches of two main profiles were present at the 21–26°2θ 02; 11 band and 30–33° 2θ 003 reflection. The high-measured intensities may be related to the high-preferred orientation (Table 4), which could not be compensated completely by the correction model. Alternatively, it may be considered that this sample has comparably low iron and high aluminum content in the octahedral sites. The coordinates derived from the Manceau et al. (2000a) study may not fit perfectly for such Al-rich varieties. The refined iron content P(Fe) = 0.65(9) was in agreement with the value of 0.695 (in the estimated confidence interval). The refined parameter P (*cis*-sites) = 1 was as expected from the results of the thermal analysis. The refined P (Cu_trien_) = 0.310(6) was lower compared with the value of 0.34 as determined by the AAM method (Table 4).

The obtained results showed that the refined *cis*-site occupancies and the iron content for purified samples were found to be comparatively consistent with the references. A tendency for underestimation of the layer charge density was observed. It may relate to the assumed values of the temperature factor. Despite the temperature factor of Cu-trien in the interlayer already having been enhanced according to Szczerba and Ufer (2018), there is still room for improvement if the temperature factor could be estimated independently, which, however, is hard to substantiate. The misfit of 001 reflection may be caused by the sample roughness, which can make deviation of peak profiles and intensities at a very low angle.

### Rietveld refinement of raw sample

The feasibility of the structural model was tested in a raw nontronite sample with natural impurities. As the Washington bulk nontronite (sample NWa) was the only one containing significant impurities, it was chosen as a representative example in this study. This raw sample was exchanged with the Cu-trien complex directly without purification and particle size-separation process.

**Figure 3:**
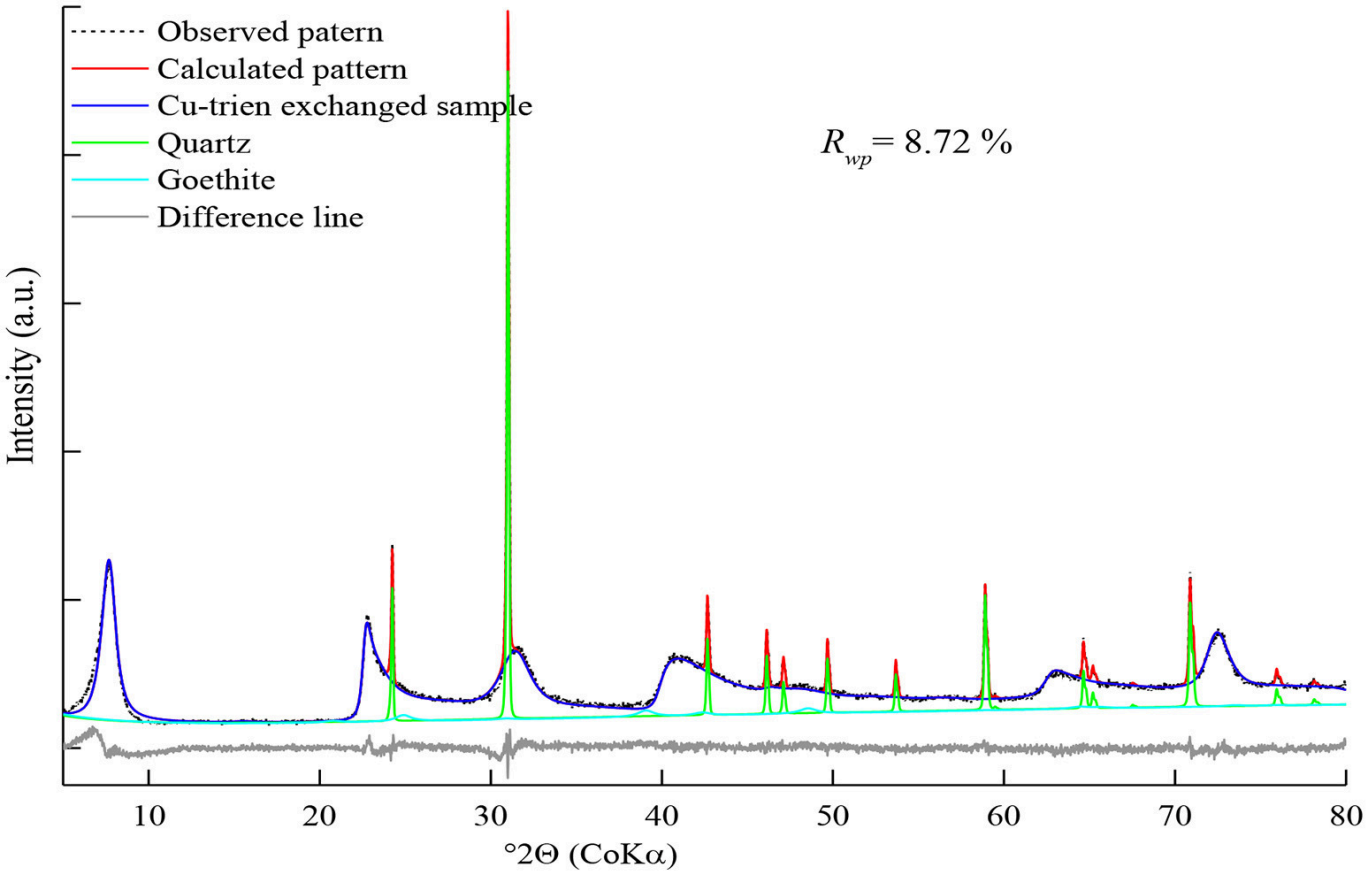
Refinement on raw Washington nontronite.

The calculated pattern was fitted satisfactorily with the observed data (Figure 3). The intensity of 02; 11 band was enhanced and matched much better than the purified sample. This might be related to the low preferred orientation (Table 5). Generally, the bulk sample had bigger particle size and less aggregation than the purified sample, which may lead to lower preferred orientation (Table 5).

**Table 5:** Comparison of the refinement results for purified and raw Washington nontronite.

	**Start value**	**Refinement limits**
			**Reference values**	**Purified sample (< 0.2 μm)**	**Raw sample (< 0.4 mm)**
				***R_*wp*_* = 9.95%**	***R_*wp*_* = 8.72%**
				***R_*exp*_* = 2.68%**	***R_*exp*_* = 3.51%**
**Contents [mass %]**					
Smectite/[3σ]				99.4(2)	79.3(4)
Quartz/[3σ]				0.1(2)	17.0(3)
Goethite/[3σ]				0.5(6)	3.7(3)
**Structure parameters**					
P(Fe)/[3σ]	0.4	0-1	0.695	0.65(9)	0.63(9)
P(*cis*-sites)/[3σ]	0.5	0-1	0.995	1	1
Layer charge density P(Cu_trien_)/[3σ]	0.2	0-0.4	0.34	0.310(6)	0.355(4)
Preferred orientation /(correction factor in < 00*l*>, deviation from random orientation)	0			1.81(3)	1.23(4)

*Reference values: P(Fe) from chemical analysis, P(cis-sites) from thermal analysis, P(Cu-trien) from layer charge density determined by the AAM method*.

The measured pattern was covered with a large number of quartz reflections and small broadened peaks of goethite. The contents of the quartz and goethite were estimated as 17.0(3) and 3.7(3)% (Table 5). The *R*_*wp*_ value of raw NWa sample decreased to 8.72%, while the refinement result of the purified sample was 9.95% (Table 5). The refined value of *cis*-octahedral site occupancy P(*cis*-sites) = 1 was in good agreement with the value of 0.995, which came from the thermal analysis (Table 1). The refined iron content P (Fe) = 0.63(9) was lower than the value of 0.695 calculated from the structural formula. However, it displayed the same tendency for the purified sample (Table 5). The refinement result of P (Cu-trien) = 0.355 (4) was slightly higher than the reference; by contrast, the refined value for the purified sample was lower than the reference (Table 5).

The refinement result of raw nontronite samples showed that the refined *cis*-octahedral site occupancies and iron content led to the same trends as the purified sample. However, the refined value of the layer charge density resulted in a reverse tendency to that of the purified sample (Table 5). This may further confirm the previous discussion: the selected coordinates may not work satisfactorily for this specific sample with lower iron-content and higher aluminum-content nontronites (Tables 4, 5). The atomic coordinates applied to all samples were chosen identically (derived by Manceau et al., 2000a); however, such a choice might be a kind of simplification or compromise.

## Summary and conclusions

The Rietveld refinements of purified Cu-trien-exchanged nontronites led to comparatively reasonable results. The refined values of the iron content and the *cis*-octahedral sites were highly consistent with the references obtained from the chemical and thermal analyses. However, the refined layer charge density showed a tendency for underestimation. It may be attributed to the uncertainty of the temperature factor of Cu-trien in the interlayer. The test of the structural model on the raw Washington nontronite showed good agreement between calculated and measured patterns. The refinement results showed similar tendency for the iron content and occupancies of *cis*-octahedral sites, but an uncertain difference of layer charge densities between the raw and purified samples. It may be a consequence of the simplification of the atomic coordinates. However, the models should be applied to more nontronite samples with different iron contents in further research so as provide evidence for this presumption.

The results of this work show that the current structure model works better for nontronites with high iron content than those with low iron content. The structure information such as the layer charge density, iron content, and occupancies of *cis*-octahedral sites can be obtained directly from X-ray diffraction patterns by the Rietveld method. This study may offer a potential for refining more structural parameters of nontronites by using this novel approach. The temperature factor of the interlayer species should be considered seriously since the occupancy of the atoms in the interlayer is related to this parameter. On the other hand, further developments should also focus on the optimization of the atomic positions, which may cover nontronite variabilities.

## Author contributions

XW performed the experiments, derived the models, interpreted the data and wrote manuscript. LL supervised development of this work, helped in data interpretation and manuscript evaluation, and acted as corresponding author.

### Conflict of interest statement

The authors declare that the research was conducted in the absence of any commercial or financial relationships that could be construed as a potential conflict of interest.
